# Sustainment of an Evidence-Based, Behavioral Health Curriculum in Schools

**DOI:** 10.1007/s11121-022-01454-5

**Published:** 2022-10-21

**Authors:** Katie Massey Combs, Karen M. Drewelow, Marion Amanda Lain, Marian Håbesland, Amy Ippolito, Nadine Finigan-Carr

**Affiliations:** 1 Institute of Behavioral Science, University of Colorado Boulder, 1440 15th St., Boulder, CO 80309 USA; 2 School of Social Work, University of Maryland, Baltimore, Baltimore, MD 21201 USA

**Keywords:** Sustainability, Evidence-based Intervention, School, Implementation Science

## Abstract

The development of evidence-based interventions (EBIs) for the prevention of behavioral health problems is well supported. However, limited data exist on the sustainability of EBIs once initial support has ceased. The current study assessed, at 2 years after initial start-up support: (1) What percent of schools sustained EBI implementation? (2) To what degree did sustaining schools implement the EBI with fidelity? (3) What were the primary reasons for not sustaining the EBI? (4) What theoretical and contextual factors during initial start-up support predicted sustainment of the EBI? The study used process evaluation data from the dissemination of the Botvin *LifeSkills Training* (LST) middle school program in 158 school districts (including 419 schools). Fifty-one percent of districts sustained the EBI and most of these sustaining districts reported following key fidelity guidelines. Primary reasons for discontinuing centered around low teacher or administrative support and turnover. Significant predictors of sustaining LST were higher ratings of LST’s complexity, benefit, and compatibility by teachers; more positive perceptions of organizational support from administrators; and smaller proportions of Black students. Results show that EBI sustainment and fidelity of implementation post-initial startup support are feasible, though likely not for all schools. Findings also suggest that cultivating support for the EBI among staff during start-up support may be helpful for sustainment and that social determinants of a school have a complex relationship with EBI sustainment. Future research should explore true causes of differences due to race/ethnicity as well as COVID-19 effects.

## Introduction

Much progress has been made in establishing evidence-based interventions (EBIs) for the prevention of behavioral health problems. Well-designed and well-implemented experimental studies have established an array of EBIs to be effective in reducing risky behaviors and maintaining results over time (Hawkins et al., 2016). As a result, governmental and philanthropic funds have been widely used to support dissemination of such EBIs. However, because data collection often ends with grant funding, information about sustainability is often unknown.

The Botvin *LifeSkills Training* (LST) Middle School curriculum is an example of an EBI that has been disseminated on a large scale. LST is a universal prevention program for middle school-age students generally facilitated by classroom teachers to promote personal self-management skills (e.g., self-esteem, problem solving, coping), social skills, and drug resistance skills (Botvin & Kantor, 2000). LST ultimately aims to reduce the long-term risks of substance use, and various randomized controlled trials demonstrate that LST decreases use of tobacco, alcohol, and marijuana up to 80%, with effects sustained through high school (Botvin et al., 1995, 2006). Furthermore, dissemination efforts have shown LST to be scalable. As of 2017, LST had been adopted by over 1200 communities serving more than one million youth with a cost benefit ratio of $13.49 for every $1 spent (Hawkins et al., 2016; Washington State Institute for Public Policy, 2019).

In addition to the field’s expanding knowledge about effective prevention programs, like LST, a body of research has explored factors that influence fidelity of implementation of EBIs delivered in community and school settings (Combs et al., 2022; Domitrovich et al., 2008; Durlak & DuPre, 2008; Harn et al., 2013; Moore et al., 2022; Nilsen, 2015). However, less evidence exists about whether such preventative behavioral health EBIs are sustained beyond initial startup funds. For example, little is known about why an EBI may not be sustained, predictors of sustainment, or how well EBIs are implemented when sustained. Sustainment is a central outcome in prevention and implementation science and it is essential to achieve population-level public health impacts (Proctor et al., 2015).

### Sustainability Theoretical Frameworks

Various definitions of sustainability exist in the EBI literature, though Scheirer and Dearing’s (2011) conceptualization has become increasingly accepted: “the continued use of program components and activities beyond their initial funding period and sometimes to continuation of desired intended outcomes” (p. 2060) (Scheirer & Dearing, 2011). As a critical outcome in implementation science, a growing number of conceptual frameworks also exist to study sustainability of interventions, many originating from the healthcare field that have since been adopted and applied to behavioral health initiatives in school and community settings (Glasgow et al., 2006; Moullin et al., 2019; Shelton et al., 2018). Several models use a comprehensive lens that also examine adoption and implementation, the stages preceding sustainability (Feldstein & Glasgow, 2008; Glasgow et al., 1999; Moullin et al., 2019; Rogers, 2003). Fewer models direct their primary focus to sustainability (Schell et al., 2013; Shelton et al., 2018) or consider the interactive and dynamic nature of the sustainability process (Chambers et al., 2013; Shelton et al., 2018). Despite variation across sustainability models, several overarching factors thought to be important to EBI sustainability emerge: contextual (e.g., policies, priorities, funding environment), organizational (e.g., leadership, stability, resources), intervention-specific (e.g., effectiveness, flexibility, complexity), and implementer-specific (e.g., acceptance, enthusiasm, skill, confidence) (Shelton et al., 2018).

This study specifically leaned on Rogers’ Diffusion of Innovations theory as a guiding conceptual model. While it primarily focuses on the adoption phase, its components have been applied to implementation and sustainment as well (Rauscher et al., 2015; Shoesmith et al., 2021). According to Rogers (2003), decisions to adopt or sustain an innovation are influenced by five factors: relative advantage (i.e., advantageous over other ideas), compatibility (i.e., consistent with current needs and values), complexity (i.e., ease of use), trialability (i.e., ability to test it), and observability (i.e., visibility of results) (Rogers, 2003).

### Evidence on Sustainment of Preventative Behavioral Health Interventions

Over the last decade, research has expanded on sustainment of health promotion interventions for youth. Four systematic reviews have examined factors associated with health behavior interventions primarily delivered in school settings (Cassar et al., 2019; Herlitz et al., 2020; Moore et al., 2022; Shoesmith et al., 2021). Cassar et al. (2019) focused on interventions promoting physical activity, synthesizing sustainability barriers and facilitators identified in seven studies. Reviewing 24 studies, Herlitz et al. (2020) also included interventions targeting additional health outcomes such as mental health, substance use, and violence. Similarly, Moore et al. (2022) reviewed 10 studies targeting mental health and well-being interventions in school settings and specifically studied barriers and facilitators to sustaining these interventions in schools. Shoesmith et al. (2021) reviewed 31 studies on a variety of health-related interventions and included two delivered in childcare settings. Although these reviews utilized varying conceptual frameworks and the interventions had different target areas, commonalities emerged with regard to barriers and facilitators influencing intervention sustainment. These themes included organizational leadership/support; staff support or belief in the intervention; resource availability (e.g., funding, staffing, infrastructure); intervention characteristics (e.g., adaptability, cost-effectiveness); implementer characteristics (e.g., motivation, confidence); and perceived need based on competing priorities (Cassar et al., 2019; Herlitz et al., 2020; Moore et al., 2022; Shoesmith et al., 2021). These systematic reviews offer important contributions on understanding the determinants of intervention sustainability in school-based settings; however, they did not screen studies to include only EBIs and largely focused on descriptions of facilitators and barriers to sustainment without examining sustainment outcomes (i.e., rates of sustainment).

Several studies have examined rates and correlates of EBI sustainment more specifically (Cooper et al., 2015; Crooks et al., 2013; McIntosh et al., 2015; Rauscher et al., 2015), though not all were implemented in school settings and several provide only descriptions (Crooks et al., 2013) or bivariate correlates of sustainment (Cooper et al., 2015; Rauscher et al., 2015). Using data from a statewide EBI dissemination initiative, Cooper et al. (2015) found that 69% of programs continued operating at least 2 years post-funding; of these, the majority was functioning at a lower level than when fully funded. Connection to a high-functioning community coalition was bivariately associated with sustainment, and issues with program “fit” (e.g., desire to change program) were bivariately associated with lower rates of sustainment. Similar studies examined correlates of sustaining among high school teachers trained in evidence-based curricula (Crooks et al., 2013; Rauscher et al., 2015). In both studies, of those who responded (slightly under half of trained teachers), 72–81% reported teaching at least “some” of the curriculum at least once. Rauscher et al. (2015) used Rogers’ diffusion of innovations as a conceptual framework and found that teachers who had more favorable attitudes about the program and who reported more experience and comfort with the program’s teaching methods had higher sustainability. No association was found between sustainability and program complexity. In Crooks et al. (2013), and similar to systematic reviews discussed above, descriptions of facilitators of sustainability included access to updated materials, training opportunities, financial resources, and support and recognition from administrators. Conversely, barriers pertained to shifts in education standards and the introduction of new programs.

In summary, despite variation in the limited studies examining sustainability of behavioral health-related programs and EBIs, a few clear themes emerge. Namely, programs can be maintained at some level beyond initial startup funds; however, lower level of functioning is common (Cooper et al., 2015; Rauscher et al., 2015). Factors that may contribute to improved sustainability outcomes include instructor’s acceptance, motivation, and comfort with delivery (Rauscher et al., 2015); logistical fit (e.g., time available); administrative support (Crooks et al., 2013; Tibbits et al., 2010); and training/technical assistance opportunities and staffing stability (Cooper et al., 2015; Crooks et al., 2013). Lastly, few studies lend insight into how contextual factors, such as the socioeconomic status (SES) or race/ethnicity of a school, affect sustainment of school-based EBIs. Though different than a curriculum-based EBI (like LST), a study on the sustainment of school-wide positive behavioral interventions and supports examined school demographic characteristics and found that they were not significant predictors of sustainment (McIntosh et al., 2015). However, school characteristics are known to be associated with implementation of an EBI (Bradshaw & Pas, 2011); thus, this area merits more investigation.

### Current Study

Several critical gaps exist in the literature that this study intends to address. First, many studies only describe reports of facilitators and barriers to sustainment (i.e., only descriptive statistics or bivariate associations) and include samples with only a portion of the schools in the original dissemination project or a portion of originally trained facilitators. Non-sustainers (versus sustainers) are generally harder to reach once initial startup funds end; thus, estimates of sustainment may be inflated and understanding of the barriers and facilitators to sustaining may unequally represent schools or communities that sustained an EBI. Second, fewer studies examine sustainability in large-scale dissemination projects reflecting more “real-world” implementation versus highly controlled trials. Finally, studies have focused on conceptual factors but not fully examined contextual variables such as race and ethnicity composition of schools, economic factors, or the effect of the Novel Coronavirus Disease 2019 (COVID-19) on sustaining. While it is known that COVID-19 had an enormous impact on schools and on behavioral health of youth (Department of Education (DOE), 2021), understanding how COVID-19 affected the sustainment of EBIs designed to mitigate behavioral health challenges is unclear. Thus, research questions were at 2 years post-initial start-up support: (1) What percent of schools sustained EBI implementation? (2) To what degree did sustaining schools implement the EBI with fidelity? (3) What were the primary reasons for not sustaining the EBI (as reported by non-sustainers)? (4) What theoretical and contextual factors (during initial start-up support) predicted sustainment?

## Method

This study used process evaluation data from a national dissemination project of the Botvin *LifeSkills Training* (LST) middle school program implemented across 15 states plus the District of Columbia. The sample includes 158 school districts (representing 419 schools) that completed a 3-year grant to support implementation of LST between academic years 2010/11 and 2018/19. All schools received pre-program training and technical assistance and implemented the EBI for at least three academic years. A university institutional review board confirmed that no ethical approval was required due to the exclusive use of retrospective data that were part of routine process evaluation.

### LST Dissemination Project

The LST middle school program is a classroom-based intervention implemented in either grades 6–8 or 7–9 aimed to reduce the long-term effects of substance use. Thirty core sessions are divided into three levels, which are taught in sequence over three school years. Level 1 consists of 15 foundational sessions that aim to build self-management, drug resistance, and general social skills. Level 2 (10 booster sessions) and level 3 (5 booster sessions) build on the skills in level 1. Additionally, the curriculum includes a total of nine optional violence prevention lessons across the three years. Each session is designed to be taught in 45–50-min classes at least once per week or up to five times per week. LST was taught by classroom teachers, though the facilitator and content area varied across schools (e.g., health, social studies, science, math, computer science, language arts, school counselors). Participating schools were provided with LST curriculum materials, training, process evaluation reports, technical assistance, and sustainability workshops at no cost. All teachers received a 1- or 2-day training workshop in the first year of implementation and were offered optional one-day booster trainings in following year(s). Training was required for all LST instructors and encouraged for school administrators and other support staff. Technical assistance was provided throughout the project, which included annual visits by consultants trained in the LST model to discuss implementation progress and problems, as well as phone-based and onsite technical assistance as needed. Finally, in an effort to build schools’ capacity to sustain LST, grant recipients were offered two regional sustainability workshops: the Training-of-Trainer workshop certified an instructor to conduct local LST trainings in their district (eliminating the expense of outside trainers after the grant) and Strategic Sustainability trainings helped districts understand the costs of LST, identify gaps in funding, and strategize ways to continue LST after completion of the grant.

### Sample

Individual or multiple schools responded to a Request for Proposal and applied to receive LST. This sample includes only schools and districts that remained in the grant for the full 3 years (88%), 158 school districts representing 419 schools. At 1- and 2-year post-grant support, an administrative staff person or the LST facilitator in each district was interviewed regarding LST sustainment. Table 1 shows descriptive statistics of school districts.

**Table 1:** Descriptive statistics of school districts by sustained and non-sustained

Variable	Total (*n* = 158)	Sustained (*n* = 81)	Not sustained (*n* = 77)	*t*-test/*χ*^2^
	M/% (SD)	M/% (SD)	M/% (SD)	
% White students	64.7 (29.8)	69.1 (28.3)	60.1(30.7)	−1.9*
% Black students	20.6 (6.9)	15.2 (22.4)	26.0 (28.7)	2.7***
% Hispanic students	9.1 (11.5)	9.7 (12.2)	8.6 (10.8)	−0.6
% Multi-race students	3.3 (2.6)	3.6 (2.7)	3.1 (2.5)	−1.3
% Asian students	1.7 (2.8)	1.7 (2.4)	1.7 (3.2)	0.1
% American Indian students	0.5 (2.1)	0.7 (2.7)	0.3 (1.0)	−1.2
% Native Hawaiian students	0.1 (0.1)	0.1 (0.1)	0.1 (0.2)	0.5
% students receiving FRL	57.0 (25.2)	53.2 (23.5)	60.8 (26.3)	1.9*
Locale–% urban/suburban	44.90%	44.40%	45.50%	0.1
Administrator organizational support	4.3 (4.4)	4.5 (0.4)	4.2 (0.6)	−2.9***
Teacher complexity, compatibility, observability	3.9 (0.6)	3.9 (0.4)	3.8 (0.4)	−2.6*
Quality of delivery	4.4 (0.4)	4.4 (0.4)	4.3 (0.4)	−0.1
COVID-affected post-grant years	57.60%	51.90%	63.60%	2.2
# of schools in district	2.7 (2.8)	2.9 (2.8)	2.4 (2.7)	−1.2

The administrator organizational support score ranged from 2.6 to 5.0; the teacher complexity, compatibility, and observability ranged from 3.0 to 4.8, and the quality of delivery score ranged from 3.2 to 5.0

* *p* < .05, *** *p* < .001

### Measures

Measures included 10 years of process evaluation data collected through classroom observations, teacher surveys, and administrator surveys during each grant-supported year, as well as publicly available data on school characteristics. Also, each district was contacted for an interview regarding sustained implementation of LST for up to 2 years following grant support. Because the dissemination project was primarily organized at the district level (e.g., schools within one district had the same coordinator), and because of limited resources to conduct individual post-grant interviews with all 419 schools, some variables were measured at the district level while others were at the school level. The headings below indicate whether a measure was collected at the district level, school level, or both.

#### Program Sustainment (School and District Level)

We used data from structured interviews with districts at 1- and 2-year post-grant to assess program sustainment. Post-grant interviews were most commonly with the district coordinator unless they had left the district; in such cases, the LST teacher(s) or other administrators participated in this interview. Interviewees were asked the number of schools currently implementing LST at an increased level, at the same level, at a reduced level, or not at all. Districts that did not respond after multiple attempts were assumed to have ceased implementation and districts that reported no implementation among any schools at the 1-year post-grant interview (and did not indicate efforts to resume in following years) were assumed to have permanently discontinued implementation, therefore they were not contacted for the 2-year post-grant interview and were coded as “not at all sustaining” given their year 1 responses. For regression analyses examining research question 4, this variable was recoded to be dichotomous indicating “sustained” (coded as 1) if the district reported sustaining LST implementation at any level among any schools or “not sustained” (0) if no schools were implementing LST at 2-year post-grant support. Sustainment was dichotomized for several reasons. First, few schools (11%) indicated implementing “at a reduced level,” and in the majority of school districts, all schools either sustained or not (i.e., districts rarely had some schools that sustained and others that did not). Thus, dichotomizing resulted in a relatively small loss of information. Second, a dichotomized sustainment outcome for inferential statistical analyses follows operationalization in prior studies on sustainment (e.g., Cooper et al., 2015; Curry et al., 2016; Rauscher et al., 2015).

#### Factors Related to Fidelity of Implementation in Sustaining Schools Post-grant Support (District Level)

The post-grant structured interview asked districts that reported any implementation about implementation. Items followed fidelity guidelines including using only facilitators who received training on the LST model, reaching all eligible students to create healthy school-wide norms, delivering the full curriculum in the prescribed order at least once per week, and using program materials (National Health Promotion Associates, 2017). Other factors related to implementation (e.g., administrative support, resources) are shown in Table 2.

**Table 2:** Descriptors of implementation of LST among sustaining school districts (*n* = 81)

	Yes (%)	Mix (%)	No (%)
Are all “required/core” lessons being taught?	66.7	14.8	18.5
Are only trained teachers implementing LST?	93.8	4.9	1.2
Are student workbooks/guides still being used?	86.4	3.7	9.9
Is LST reaching ALL students in the 3 grade levels?	48.1	23.4	28.6
Is LST adequately supported at the administrative and teacher level?	81.5	3.2	12.3
Is there a person responsible for oversight and coordination of LST?	70.4	0.0	29.6
Is fidelity being monitored?	29.6	0.0	70.4
Is the TOT certified during the grant still onsite?	67.9	21.0	11.0
• If yes, has the TOT provided any training since the grant ended? (*n* = 69)	31.9	10.1	58.0
Is there funding next year for … (*n* = 80)			
• Student workbooks?	40.0	0.0	60.0
• Manuals for new teachers?	40.0	0.0	60.0
• Training new teachers?	41.3	0.0	58.8
• Paying substitutes for trainees?	38.8	0.0	61.3
Is LST supported financially or in other ways by a community coalition or agency? (*n* = 80)	18.8	0.0	81.3

Trainer of trainers (TOT)

“Mix” indicates that some schools in a district, but not all, indicated yes to the question

#### Reasons for Not Sustaining (School Level)

The post-grant structured interview asked districts, “for every school that discontinued, what was the primary reason and, if applicable, the secondary reason for discontinuing?” The interviewee provided open-ended responses, which were then coded into the following 11 options: *time constraints (or other academic priorities), scheduling and placement issues, prefer to use health curriculum or other program, lack of teacher buy-in, teacher turnover, lack of administrative support or administrative turnover, lack of funding for materials or training, fidelity monitoring was burdensome, overall disorganization, unknown, or other.*

#### Predictor Variables for Sustainment (District Level)

Predictors for the logistic regression (i.e., research question 4) included variables that were measured during grant-supported implementation, thus measured prior to the post-grant interview. These included observer-reported quality of delivery; an overall score of teachers’ perceptions of LST’s complexity, benefit, and compatibility; administrators’ perceptions of organizational support; districts with post-grant years during COVID-19; and school characteristics (i.e., locale, percent of students receiving free and reduced lunch (FRL), and racial and ethnic makeup of schools).

##### Facilitators’ Quality of Delivery During Grant Support

Quality of delivery is a common domain of fidelity of implementation (Durlak & DuPre, 2008). Given our relatively small sample and need for a parsimonious model, we chose this as the single fidelity of implementation predictor because research supports that domains like quality of delivery, reflecting how well an EBI is implemented, are more closely linked to student outcomes than domains like adherence or dosage, reflecting the amount of an EBI that students received (Durlak & DuPre, 2008). Quality of delivery was measured through seven items assessing the instructor’s delivery of lessons and was reported by local observers during classroom observations. Items included the teacher’s knowledge of the program, enthusiasm, poise and confidence, rapport and communication, classroom management, ability to address questions, and overall quality of the lesson. Response options were on a Likert scale (1 = poor to 5 = excellent) and had strong internal reliability (*α* = 0.95). The seven items were averaged to create a mean quality of delivery score.

##### Administrators’ Report of Organizational Support

At the end each grant-supported year of LST implementation, district coordinators completed a survey regarding implementation, organizational characteristics, and administrative support. Given prior research findings that elements of organizational support are predictive of sustaining (Cooper et al., 2015; Herlitz et al., 2020), we created a mean score reflecting district-level administrative and organizational support. The items began with the prompt: “Throughout the implementation process, how much have each of the following been an asset or a barrier: administrative support and leadership (moral support); buy-in/support of LST at district level; buy-in/support of LST at principal level; fit between LST and other school programs and goals; key staff participation in planning, decision making, and problem solving; cohesiveness and collaboration among all key stakeholders in program; program coordinator or champion of the program” (response options: 1 = significant barrier, 5 = significant asset). Items were averaged to create the administrative/organizational support score for each district (*α* = 0.94).

##### Facilitators’ Perceptions of EBI’s Complexity, Benefit, and Compatibility

Following LST implementation in each grant-supported year, facilitators were invited to complete a survey. Using Roger’s diffusion of innovations, we created a variable reflecting three of Roger’s key concepts: perceived complexity, observability, and compatibility (items reflecting trialability and relative advantage were not available). Five items reflected complexity and asked for teachers’ overall rating (1 = poor, 5 = excellent) of time required to implement LST, ease of implementation, quality of materials, training, and program flexibility. Two items reflected observability and included: “the LST program teaches students the skills needed to avoid drugs and violence,” and “the LST program has the potential to play a significant role in reducing youth participation in drugs” (1 = strongly disagree, 5 = strongly agree). Three items reflected compatibility: “I am in favor of having the LST program in my school;” “Parents were supportive of their children’s participation in the LST program;” and “school administrators were supportive of the LST program” (1 = strongly disagree, 5 = strongly agree). Though we intended to create individual constructs for each key concept, they were highly correlated (*r* > .75), and thus to avoid multicollinearity and to create a parsimonious model, we used a global score reflecting the three concepts from Roger’s theory of diffusion of innovations (10 items, *α* = .94).

##### COVID-19-Affected Post-grant Years

While the majority of districts received 3 years of grant support, 20% of districts received one or more additional year(s) of minimal support (e.g., training and curriculum materials) based on need and commitment during the grant. For districts entering a 3-year grant cycle between 2010 and 2013, grant support concluded no later than Spring 2016, and the two post-grant sustainability interviews were completed as late as the 2016–2017 and 2017–2018 academic years. A subsequent grant cycle started in Fall 2016 and concluded in Spring 2019, whereby sustainability post-grant interviews occurred in the 2019–2020 and 2020–2021 academic years. The COVID-19 pandemic heavily impacted U.S. school operations starting in March 2020; as a result, this sample naturally had a clean split between districts whose post-grant years fell prior to COVID-19 and whose post-grant years fell during COVID-19. Among this sample, 67 districts’ first two post-grant years fell before 2019, and 91 districts post-grant years were in the COVID-19-affected 2019–2020 and 2020–2021 academic years. A variable was created to indicate COVID-19-affected (1) and unaffected districts (0).

##### Characteristics of School Districts

Demographics of school districts were included to assess how the context of SES, racial/ethnic makeup, and locale may affect sustainability. These characteristics reflect widely documented disparities in education and health (Finigan-Carr, 2017), but have not thoroughly been explored in studies assessing sustainability of an EBI (McIntosh et al., 2015). The percentage of students receiving FRL was used as a proxy for SES of the district. Race/ethnicity included the percent of students who identified as White, Black, Hispanic, Asian, American Indian, and Native Hawaiian. School district demographics were collected from the National Center for Education Statistics (NCES) and schools’ data were aggregated to the district level. Locale was also collected from NCES and recoded into “mostly rural” (0) and “mostly suburban/urban” (1) at the district level.

### Analysis

Frequencies were used to describe the proportion of school districts (and individual schools) that sustained LST (i.e., research question 1), reasons that schools did not sustain LST (i.e., research question 2), and fidelity of implementation among sustaining schools (i.e., research question 3). For research question 4, the association between sustaining LST and each predictor variable was first explored through bivariate analyses of chi-square and *t*-tests (Table 1). Then, a binary logistic regression was conducted with program sustainment as the dependent variable. Due to the high correlation between the percentage of students receiving FRL and the percentage of students who identified as Black and White (*r* > .6), we examined models using FRL or race and ethnicity. The model using race and ethnicity explained more variance and had stronger model fit statistics compared to the model with FRL only; thus, we excluded FRL. There were no missing data at the district level. Three schools had missingness on FRL and race/ethnicity; these schools’ race/ethnicity and FRL data were not included in their district average.

## Results

### Research Question 1: Program Sustainment

Of the 158 school districts, 51% (*n* = 81) sustained LST in at least one participating school at 2-year post-grant support. At the school level, of the 419 individual schools, 30% (*n* = 125) reported implementing at the same level (or higher), 11% (*n* = 46) at a reduced level, and 59% (*n* = 248) not at all. Schools within a district generally either all sustained or all discontinued by 2-year post-grant support, with the main reason being that a large proportion of districts (*n* = 78) were comprised of only one school. Of the 158 districts, only 16% (*n* = 25) had a mix of schools that sustained and discontinued by 2-year post-grant support.

### Research Question 2: Factors Related to Fidelity of Implementation 2 Years Post-grant

Table 2 shows district-reported indicators from the 81 sustaining districts describing their LST implementation. Two-thirds (67%) of districts reported that schools were delivering all core lessons, 94% used only LST-trained teachers, and about three-quarters (72%) were reaching all eligible students in one or more schools. Furthermore, 68% had a “trainer of trainers” onsite, and 51% (*n* = 41) had funding for at least one program component. Additionally, 82% reported support from administration and teachers, 30% monitored fidelity, and 19% received outside support.

### Research Question 3: Reasons for Not Sustaining

Table 3 presents reasons reported for not sustaining among the 248 non-sustaining schools. In total, 430 primary and secondary reasons were reported for the 248 non-sustaining schools. The most common primary reasons were time constraints/other academic priorities (16.9% of schools), teacher turnover (16.9%), lack of administrative support/turnover (16.1%), and preference for another health curriculum/other programs (16.1%). The major secondary reasons for not sustaining were similar to those listed in the primary reasons.

**Table 3:** Primary reasons schools reported for not sustaining the EBI (*n* = 248)

Reason reported for not sustaining EBI	Primary reasons, *n* = 248	Secondary reasons, *n* = 182	Total reasons, *n* = 430
Time constraints/other academic priorities	16.9%	9.3%	26.2%
Scheduling and placement issues	7.3%	8.1%	15.3%
Preference for another curriculum/program	16.1%	8.5%	24.6%
Lack of teacher buy-in	9.7%	15.7%	25.4%
Teacher turnover	16.9%	4.0%	21.0%
Lack of admin support or admin turnover	16.1%	9.7%	25.8%
Lack of funding for materials/training	3.2%	7.3%	10.5%
Fidelity monitoring burdensome	0.0%	0.0%	0.0%
Overall disorganization	0.4%	3.6%	4.0%
Unknown/other	13.3%	7.3%	20.6%

Every school that was not sustaining had a “primary reason”; thus, percentages for primary reasons add up to 100%. Of the 248 non-sustaining schools, 182 schools also reported a secondary reason for not sustaining. Because of this, percentages in the secondary column equal less than 100% and percentages in the total equal greater than 100%

### Research Question 4: Predicting Program Sustainment

Table 1 shows bivariate associations between each predictor and program sustainment. In the logistic regression (Table 4), significant predictors (at *p* < .05) of sustaining LST 2 years post-grant support were higher ratings of LST’s complexity, benefit, and compatibility by teachers; more positive perceptions of organizational support from administrators; and districts that had smaller proportions of Black students. For every one-point increase in teachers’ perceptions and in administrative support during grant-supported implementation, the school district had, respectively, 3.5 and 2.7 greater odds of sustaining.

**Table 4:** Logistic regression predicting program sustainment (*N* = 158)

	B	S.E	Sig	Exp(B)	95% C.I. of Exp(B)	
					Lower	Upper
COVID-affected post-grant years (1)	−0.73	0.39	0.06	0.48	0.23	1.04
Teacher complexity, compatibility, observability	1.25	0.54	0.02	3.50	1.21	10.10
Administrator organizational support	0.98	0.37	< 0.01	2.65	1.28	5.48
Quality of delivery	0.18	0.46	0.70	1.19	0.48	2.97
% Black students	−0.02	0.01	< 0.01	0.98	0.96	0.99
% Hispanic students	0.03	0.02	0.09	1.03	1.00	1.07
% Multi-race students	0.08	0.07	0.26	1.09	0.94	1.25
% American Indian students	0.08	0.13	0.55	1.08	0.84	1.39
Locale: suburban/urban (1)	−0.35	0.42	0.41	0.71	0.31	1.61
# of schools in district	0.14	0.08	0.07	1.15	0.99	1.33
Constant	−9.76	3.26	< 0.01	0.00		

## Discussion

Among the 158 school districts that received 3 years of supported implementation, 51% sustained the EBI in at least one participating school; among these sustaining districts, the majority reported following key fidelity guidelines (e.g., teaching all core lessons, only using trained teachers, using EBI materials, reaching all students). At the school-level (including 419 schools), roughly 40% of individual schools sustained. Previous studies in a variety of community and school settings reported higher rates of sustaining a behavioral health intervention (i.e., 60–80% sustainment rates) (Cooper et al., 2015; Curry et al., 2016; Rauscher et al., 2015; Tibbits et al., 2010). Higher sustainment in other studies is likely related to lower response rates, less stringent definitions of sustaining, and study populations outside of schools.

First, samples in prior studies of EBI sustainment represented between 45 and 79% of facilitators or programs originally included in the dissemination project (or originally trained), whereas the present study includes all school districts/schools that remained in the 3-year dissemination project (i.e., 100% response rate). It is likely that sustainment rates in other studies (Cooper et al., 2015; Curry et al., 2016; Rauscher et al., 2015; Tibbits et al., 2010) are limited by some degree of response bias in which teachers or schools who responded were also more likely to have sustained. Second, the varying thresholds for “sustained” may contribute to a lower sustainment rate. In Cooper et al. (2015), schools were considered sustainers if they incorporated “bits and pieces” of the EBI, and in Rauscher et al. (2015), teachers were considered sustainers if they taught a single lesson once over the next year. Such cases were not considered sustaining in the current study. While we considered “reduced implementation” as sustaining, this designation required teaching more than one full lesson and an attempt to reach most eligible students. Third, the setting or the target population likely impacts sustainment rates. Schools are large systems that over 2 years can undergo significant changes in administration, teachers, and priorities. In fact, in Cooper et al. (2015), EBI sustainment differed by type of program, with only 31% of classroom-based (i.e., school) programs sustaining versus 75% of family-focused prevention programs. Sustaining a preventative behavioral health EBI in a school is likely more challenging, particularly for universal school-wide interventions that require system-wide cooperation.

In the 81 districts (51%) that sustained LST, fidelity of implementation appeared strong with at least two-thirds of districts reporting adherence to various recommendations for fidelity of implementation. Two-thirds of districts reported all core lessons were taught, almost all schools (> 80%) reported using only trained teachers and LST student workbooks, and about half was still reaching 100% of eligible students. Considering the various factors that make schools a challenging environment for sustaining an EBI, these reports of implementation appear strong, especially given that many districts had limited implementation support or funding assistance. While one study evaluated fidelity of implementation after initial startup funds through dosage (i.e., how much of the program was delivered), other domains of fidelity of implementation have been largely unexplored (Rauscher et al., 2015). These data are therefore significant contributions given the scarcity of data on implementation after initial start-up support.

In all analyses, the value of teacher and administrative support was clear. For the 248 schools that discontinued LST, lack of teacher or administrative support and turnover reflected 43% of the primary reasons for not sustaining; in the logistic regression predicting sustainment, teachers’ perceptions of LST and administrative support during implementation were strong predictors of sustaining 2 years later. These findings confirm previous studies noting the importance of these factors (e.g., Tibbits et al., 2010), which are also reflected in theoretical frameworks (Rogers, 2003). To cultivate support, “championing” the program both within the school setting and beyond can be invaluable. Gathering student, teacher, and administrator feedback throughout implementation, conveying results and highlighting successes building-wide has the potential to maintain and promote support (McIntosh et al., 2015). Indeed, broad communication about the EBI builds awareness, invites other personnel to reinforce concepts, and provides continuity during turnover. Beyond the school, parent, and community awareness of programming, and related outcomes, can provide opportunities to convey feedback about the value of the EBI as well as provide material and immaterial support for sustaining programming.

Perhaps surprisingly, funding was rarely mentioned as a reason for not sustaining and only half of sustaining districts had any funding support. Insufficient funding has been noted as a barrier to sustaining in prior studies (Herlitz et al., 2020), and our finding may be unique to this dissemination project as it was designed to help build schools’ capacity to sustain LST with minimal ongoing funding. Indeed, the two primary expenses related to program sustainability are teacher training and student guides. During the grant, teacher training and technical assistance were provided, as well as the option to attend a Training-of-Trainers workshop to conduct local teacher training and Strategic Sustainability Workshops to discuss funding strategies.

This study also examined contextual factors of a school district as predictors of sustaining in the logistic regression analysis. One key contextual factor potentially important to sustainability are school demographics, particularly those related to socioeconomic status and racial and ethnic composition of the student body (Bradshaw & Pas, 2011; Forman et al., 2013; McIntosh et al., 2015). Racial and ethnic composition was a significant predictor of sustaining LST, specifically the proportion of Black students in a district was negatively associated with sustaining. However, the relationship between the proportion of Hispanic students in a district and sustaining was positively associated, though not statistically significant (*p* = .09). While not included in the regression due to multicollinearity with racial and ethnic categories, a discussion of SES and its effect on intervention sustainability is warranted. The proportion of students receiving FRL within a district, used as a proxy for SES, was positively correlated with the proportion of Black students, while negatively correlated with the proportion of White students (*r* = .63). Correlations between FRL and other racial and ethnic groups were lower (e.g., *r* < .3 for Hispanic, multi-race, and Asian). This may speak to the notion that these associations between race and sustainment in the current study actually reflect SES due to racism and structural factors related to school funding in neighborhoods disproportionately impacted by low SES (Blanchett et al., 2005). Researchers have demonstrated that the availability of resources in schools is associated with school funding inequities along racial and ethnic lines (Blanchett et al., 2005; Necochea & Cline, 1996). Thus, the strong inverse relationship between the proportion of Black students and sustainment is likely a marker of community SES and resources in mostly White versus mostly Black school districts. Ultimately, the racial and ethnic composition of a district appears to have a complex relationship with sustaining a school-based, behavioral health EBI. Such findings highlight the complexity of these constructs when used as predictors, the importance of thorough racial and ethnic categories, and the need for future research.

### Future Directions and Limitations

Findings warrant future research on the impact of COVID-19 on implementation and sustainment of EBIs. COVID-19 had a negative association with sustaining though not quite statistically significant (*p* = .06). Throughout the pandemic, schools had to redirect attention to navigating logistics of schooling (e.g., virtual, hybrid), turnover of administrators and teachers, and safety of the school (Department of Education (DOE), 2021). This likely limited their ability to commit to LST, even if they felt a great need for it. As students have faced many challenges of a global pandemic (e.g., illness or death of a loved one, abrupt school shutdowns) (Department of Education (DOE), 2021), sustaining programming that is mentally and emotionally supportive will be tremendously beneficial.

Several significant limitations must be noted. First, while our logistic regression model maintained temporal order (i.e., predictors were all measured prior to the outcome), this research is correlational and, as such, findings indicate statistical associations and do not establish causality. Also, schools in this project received ample implementation support for 3 years, and thus, results likely are not representative of schools that adopt the program on their own or through a project providing fewer implementation and sustainment supports. Due to how the dissemination project and process evaluation was organized, many data were collected at the school district level, specifically data for the logistic regression predicting sustainment and data on fidelity of implementation post-grant support. These analyses combined 419 schools into 158 school districts, which may have conflated and inadequately summarized school-level predictors. Finally, in a few cases, project coordinators made decisions on why a school did not sustain, allowing an opportunity for bias and variability into the collection of these data. Generally, these cases were coded as “other/unknown,” except when a firm and informed decision could be made.

## Conclusions

In this sample, half of school districts sustained the EBI 2 years post-start-up support and reported following major fidelity guidelines. Teachers’ perceptions of the EBI (reflecting Roger’s key concepts) and administrator support emerged as the critical factors that increased sustainment and suggests that cultivating support for the EBI among staff during start-up support may be helpful for sustainment. The complex relationships between race and ethnicity, FRL, and sustainment provides a glimpse into the impact of these important socioeconomic constructs and social determinants; it is important to ascertain which racial/ethnic categories should be used in implementation research and to further investigate the true causes of differences noted due to race and ethnicity. Future research should explore these factors as well as COVID-19 effects systematically as they pertain to EBI sustainment. Finally, as the field of prevention science partners with schools and communities (who are experts of their schools and are navigating many important outcomes), we must acknowledge that sustaining any specific EBI is not always the “right” choice for some schools given their specific circumstances.

## Data Availability

Available from authors upon request.
